# Comparative efficacy and safety of mesalazine-based regimens with traditional Chinese medicines in mild-to-moderate ulcerative colitis

**DOI:** 10.3389/fphar.2025.1691460

**Published:** 2026-01-13

**Authors:** Bin Huang, Honglin An, Liming Chen, Huimin Lin, Huaping Wu, Ruofei Li, Yaping Su, Jiumao Lin, Dan Shi

**Affiliations:** 1 Academy of Integrative Medicine, Fujian Key Laboratory of Integrative Medicine on Geriatrics, Key laboratory of Integrative Medicine of Fujian Province University, Fujian University of Traditional Chinese Medicine, Fuzhou, Fujian, China; 2 The Second Affiliated Hospital of Fujian University of Traditional Chinese Medicine, Fuzhou, Fujian, China; 3 The Third People’s Hospital of Fujian Province, Fuzhou, Fujian, China; 4 Fujian Maternity and Child Health Hospital, Fuzhou, Fujian, China

**Keywords:** ulcerative colitis, mesalazine, traditional Chinese medicine, network meta-analysis, TNF-α

## Abstract

**Background:**

Traditional Chinese medicine (TCM) formulations are increasingly used in combination with mesalazine to treat mild-to-moderate active ulcerative colitis (UC). However, direct comparisons between various TCM regimens are limited.

**Methods:**

We performed a frequentist network meta-analysis of 34 randomized controlled trials (n = 2,854) comparing oral mesalazine (1.0–4.0 g/day) alone versus mesalazine plus one of eight TCM formulations: Kangfuxin solution, Shaoyao decoction, Glycyrrhizae decoction, Scutellaria decoction (Huangqin granules), Baitouweng decoction (Pulsatilla; retention enema), Shenling Baizhu Powder, CurQD formula, or Fufangkushen capsules. Outcomes included clinical efficacy, adverse events, Mayo score, serum interleukin-6 (IL-6) and tumor necrosis factor-α (TNF-α), and intestinal Bifidobacteria, Lactobacilli, and *Escherichia coli*. Risk ratios (RRs) were calculated for dichotomous outcomes and mean differences (MDs) for continuous outcomes. Treatments were ranked using surface under the cumulative ranking curve (SUCRA).

**Results:**

Most TCM–mesalazine combinations improved clinical efficacy versus mesalazine alone. CurQD and Kangfuxin had the highest probabilities of being most effective for symptom improvement (SUCRA 96.7% and 72.7%, respectively); the direct CurQD–mesalazine comparison showed RR = 2.67 (95% CI 1.16–6.14). Adverse-event rates were similar across regimens, with lower incidence of adverse events. Mayo score reductions were greatest with Glycyrrhizae decoction (MD = −1.40), Baitouweng decoction via retention enema (MD = −1.09), and Kangfuxin solution (MD = −1.07). Scutellaria granules produced the largest IL-6 decrease (MD = −53.28 pg/mL) and ranked highest for TNF-α reduction, followed by Kangfuxin. For gut microbiota, Shaoyao decoction ranked highest for increasing Bifidobacteria, Glycyrrhizae decoction for increasing Lactobacilli, and Glycyrrhizae also reduced *E. coli* (MD = −1.93).

**Conclusion:**

Combining mesalazine with selected TCM formulations can enhance clinical response, reduce inflammatory cytokines, and beneficially modulate gut microbiota without increasing adverse events. CurQD or Kangfuxin may be prioritized for symptomatic improvement, Glycyrrhizae or Baitouweng for Mayo score reduction, Scutellaria for cytokine control, and Shaoyao or Glycyrrhizae for microbiota modulation. High-quality multicenter RCTs are warranted to confirm these comparative rankings.

## Introduction

Ulcerative colitis (UC) is a chronic, relapsing inflammatory bowel disease characterized by diffuse mucosal inflammation confined to the colon and rectum (Pang, 2023; Yang et al., 2023). Its incidence and prevalence have steadily increased worldwide over the past 2 decades, including in traditionally low-incidence regions such as East Asia (Levine et al., 2020). The disease imposes a substantial burden on patients, with symptoms such as persistent diarrhea, abdominal pain, rectal bleeding, and fatigue contributing to impaired quality of life, reduced work productivity, and psychological distress (Wakai et al., 2020). UC is associated with long-term risks including colorectal dysplasia and cancer, particularly in patients with prolonged disease duration and extensive colonic involvement (Wan et al., 2021; Yue et al., 2025). Mesalazine (5-aminosalicylic acid) remains the cornerstone of pharmacologic therapy for mild-to-moderate UC, particularly in inducing and maintaining remission (Lima et al., 2022). However, despite its widespread use and established anti-inflammatory effects, the therapeutic response to mesalazine varies considerably among patients. Issues such as incomplete symptom resolution, frequent relapse, suboptimal adherence due to dosing frequency or gastrointestinal side effects, and concerns over nephrotoxicity in long-term use highlight the limitations of monotherapy (Martí-Aguado et al., 2021; Lu et al., 2024). These shortcomings necessitate adjunctive strategies to enhance treatment outcomes and reduce drug burden.

Traditional Chinese medicine (TCM) has been increasingly integrated into UC management, particularly in East Asia, owing to its multi-targeted effects on immune modulation, inflammation control, and mucosal repair. Several TCM formulations have demonstrated promising effects in alleviating UC symptoms, lowering systemic and local cytokine levels, and promoting intestinal microbiota balance in both clinical and preclinical studies. Given these potential benefits, there is growing interest in combining TCM with mesalazine to enhance therapeutic efficacy and safety in UC patients (Kou et al., 2020; Shen et al., 2021). Over the past decade, an increasing number of randomized controlled trials (RCTs) have investigated the efficacy of various TCM formulations combined with mesalazine in the treatment of UC. Combinations such as Kangfuxin solution, Shaoyao Decoction, Glycyrrhizae decoction, and Baitouweng Decoction with mesalazine have shown favorable outcomes in improving clinical remission rates, reducing inflammatory cytokines (e.g., IL-6, TNF-α), and modulating gut microbiota (Chen, 2018; Kong et al., 2025). These findings suggest that TCM may provide an effective adjunct to standard mesalazine therapy (Yang et al., 2023; Zhang et al., 2022).

However, most of the existing evidence comes from small-scale two-arm RCTs with varied diagnostic criteria, outcome measures, and sample sizes. Head-to-head comparisons of different TCM–mesalazine combinations are scarce, and long-term outcomes or adverse event profiles are often underreported. Such methodological inconsistencies limit the generalizability and interpretability of these findings, hindering the evidence-based integration of TCM into standardized UC management. To date, no comprehensive network meta-analysis has systematically compared the efficacy and safety of various TCM formulations in combination with mesalazine, leaving a critical gap in the selection of optimal treatment strategies for integrative care.

Despite the potential benefits of TCM in the treatment of UC, there is a lack of systematic comparative studies to determine which TCM formulations work best in combination with mesalazine. Therefore, this study aims to perform a network meta-analysis to compare the efficacy and safety of different TCM–mesalazine combinations. In response to this gap, we conducted a frequentist network meta-analysis that included 34 RCTs evaluating eight TCM–mesalazine combinations for active mild-to-moderate UC. This analysis allowed both direct and indirect comparisons, enabling us to rank the interventions based on their efficacy and safety using surface under the cumulative ranking curve (SUCRA) probabilities. We specifically selected TCM formulations that (i) had randomized trials directly comparing “TCM + mesalazine” versus “mesalazine alone,” ensuring a connected network for quantitative synthesis; (ii) represent complementary pharmacological actions relevant to UC, such as NF-κB–mediated cytokine suppression (e.g., Scutellaria granules, CurQD), mucosal healing (e.g., Kangfuxin), microbiota and barrier modulation (e.g., Shaoyao and Glycyrrhizae decoctions), topical therapy for distal disease (Baitouweng retention enema), and broad immune support (Shenling Baizhu Powder); (iii) are available as fixed-composition, clinically used preparations (decoctions/granules/capsules/liquids) to ensure reproducibility; and (iv) can be co-administered with mesalazine in routine care. The final set of TCM formulations included Kangfuxin solution, Shaoyao decoction, Glycyrrhizae (Gancao Xiexin) decoction, Scutellaria (Huangqin) granules/decoction, Baitouweng decoction (retention enema), Shenling Baizhu Powder, CurQD, and Fufangkushen capsules. Although CurQD and Fufangkushen were each represented by a single RCT, they were retained due to their clinical relevance, and sensitivity analyses were conducted to assess the robustness of the rankings excluding single-trial interventions. This study provides a comprehensive, quantitative synthesis of the efficacy and safety of TCM–mesalazine combinations, offering valuable insights for individualized UC treatment strategies.

## Methods

### Literature search strategy

We performed a comprehensive literature search across five major databases: PubMed, China National Knowledge Infrastructure (CNKI), Wanfang Data, VIP Database, and SinoMed. The search spanned from database inception to July 2025. Both Medical Subject Headings (MeSH) and free-text keywords were used in combination to maximize sensitivity and specificity. English search terms included UC, mesalazine, TCM, Shaoyao decoction, Kangfuxin solution, and randomized controlled trial. Equivalent Chinese terms were applied to Chinese-language databases to capture regionally published trials. Boolean operators (AND, OR) were used to structure the queries. No restrictions were applied to publication language; however, only studies published in English or Chinese were eligible. Reference lists of all included articles and relevant reviews were also manually screened to identify additional studies missed by the electronic search.

### Eligibility criteria

Studies were included if they met all of the following criteria:

1. Study design: Randomized controlled trials (RCTs), regardless of blinding or allocation concealment status.
2. Population: Patients with a confirmed diagnosis of active mild-to-moderate UC, based on established clinical, endoscopic, and/or histopathological criteria.
3. Intervention: The experimental group received a single TCM formula—either a classical decoction or Chinese patent medicine—administered orally or via enema, in combination with oral mesalazine. The control group received oral mesalazine monotherapy.
4. Outcomes: Studies were required to report at least one of the following outcomes: overall clinical efficacy rate, Mayo score, levels of inflammatory cytokines (IL-6, TNF-α), intestinal microbiota indices (e.g., Bifidobacteria, Lactobacilli, *Escherichia coli*), or incidence of adverse events.

Studies were excluded if they met any of the following conditions:

1. Use of more than one TCM formulation, or concurrent use of non-pharmacologic traditional therapies such as acupuncture or moxibustion in the experimental group.
2. Non-randomized trials, quasi-experimental studies, or trials with major methodological flaws.
3. Incomplete or irretrievable outcome data, or studies that failed to report extractable quantitative results.
4. Duplicate publications or abstracts without corresponding full texts.

### Data extraction

Data from eligible studies were independently extracted by two reviewers using a pre-defined standardized data collection form. Extracted information included: first author, year of publication, country or region where the study was conducted, sample size in each group, baseline characteristics of participants (mean age, sex distribution), details of interventions (name and dosage of the TCM formulation, mesalazine regimen, route of administration), treatment duration, and all reported outcome measures. These included both primary outcomes (e.g., clinical efficacy rate, Mayo score) and secondary outcomes (e.g., serum or mucosal levels of IL-6, TNF-α; intestinal microbiota changes; adverse event rates). Any discrepancies between the two reviewers were resolved by discussion, and if consensus could not be reached, a third reviewer was consulted to adjudicate.

#### Risk of bias assessment

The methodological quality of the included randomized controlled trials was evaluated using the Cochrane Risk of Bias Tool (RoB 2.0). This tool assesses seven domains: (1) random sequence generation, (2) allocation concealment, (3) blinding of participants and personnel, (4) blinding of outcome assessment, (5) completeness of outcome data, (6) selective outcome reporting, and (7) other potential sources of bias (Jang et al., 2021). Assessments were visualized using RevMan 5.4.

### Statistical analysis

All statistical analyses were performed using Stata (StataCorp, College Station, TX, United States). For dichotomous outcomes, effect sizes were calculated as risk ratio (RR) with 95% confidence intervals (CIs). For continuous outcomes, mean difference (MD) with 95% CIs. We conducted a frequentist random-effects network meta-analysis (NMA) integrating direct and indirect comparisons. Surface under the cumulative ranking curve (SUCRA) values were calculated; higher SUCRA indicates higher probability of being best.

Heterogeneity across studies was assessed using the *I* ^*2*^ statistic. To detect potential publication bias, comparison-adjusted funnel plots were visually inspected, and asymmetry was formally tested using Egger’s regression. Inconsistency between direct and indirect evidence was evaluated using the node-splitting method when closed loops were present in the treatment network. A *p*-value <0.05 was considered statistically significant for all analyses.

## Results

### Study selection and characteristics

**FIGURE 1:**
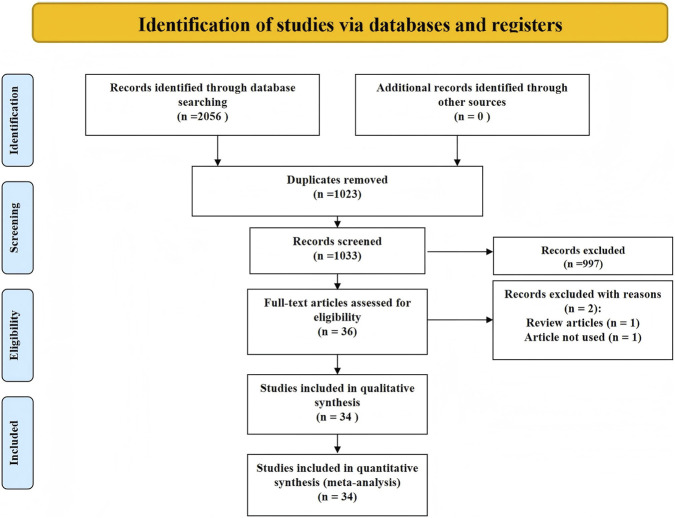
PRISMA flow diagram of study selection.

A total of 2,056 records were identified. After removing 1,023 duplicates, 1,033 titles/abstracts were screened and 36 full texts were assessed. Two full texts were excluded (one review; one not meeting eligibility), leaving 34 RCTs for the network meta-analysis (Chen, 2018; Kong et al., 2025; Liu, 2019; Ben-Horin et al., 2024; Zheng et al., 2015; Shen, 2021; Li et al., 2024; Feng, 2023; Ding et al., 2018; Wang and Zhang, 2024; Chen et al., 2019; Jia-Ping and Chao-Qun, 2019; Yang and Wang, 2015; Chen, 2022; Zhu and Ji, 2022; Shan and Chen, 2022; Liu L., 2022; Xu et al., 2022; Liu Y., 2022; Yang and Yan, 2018; Dong, 2018; Li and Wang, 2021; Li, 2015; Wei, 2013; Chen, 2014; He, 2014; Chen et al., 2013; Huang, 2016; Zhang, 2019; Gong et al., 2012; Hu, 2025; Liu, 2024; Meng, 2025). The literature selection process is illustrated in Figure 1.

All trials were conducted in China except one multicenter RCT from Israel (CurQD). Sample sizes ranged from 41 to 314; mean ages ranged from approximately 20–56 years. Treatment duration was 3–12 weeks. Controls received oral mesalazine monotherapy (1.0–4.0 g/day). Experimental arms combined mesalazine with one TCM: Shaoyao decoction (n = 3), Glycyrrhizae decoction (n = 5), Scutellaria decoction (n = 2), Baitouweng decoction (n = 4; retention enema), Kangfuxin solution (n = 10), Shenling Baizhu Powder (n = 8), CurQD formula (n = 1), or Fufangkushen capsules (n = 1) (Table 1).

**TABLE 1:** Characteristics of the 34 included randomized controlled trials.

No.	Author (year)	Study design, region	Sample of study	Age		Sex (M/F)		Interventions		Severity of ulcerative colitis	Duration (Weeks)	Outcomes
				Control group	Experimental group	Control group	Experimental group	Control group	Experimental group			
1	Chen Jianlin (2018)	RCTs, China	72	37.9 ± 8.66	38.5 ± 8.78	21/15	20/16	Mesalazine enteric-coated tablets, 0.5 g per dose, three times daily, orally	On the basis of oral mesalazine enteric-coated tablet treatment, shaoyao decoction retention enema was additionally administered	In the treatment group, there were 16 cases of mild, 18 moderate, and 2 severe ulcerative colitis. In the control group, there were 18 mild, 17 moderate, and 1 severe case	3 weeks	Overall clinical efficacy, serum inflammatory factor levels, intestinal microbiota levels
2	Liu Yanhong (2019)	RCTs, China	81	39.46 ± 6.38	40.15 ± 5.27	23/17	24/17	Mesalazine enteric-coated tablets (NMPA H19980148), oral administration: 0.5 g (2 tablets) per dose, 3 times daily	Mesalazine enteric-coated tablets combined with modified shaoyao decoction retention enema therapy	The disease duration in the control group was 2–4 years (3.17 ± 1.02), and in the treatment group was 2–5 years (3.46 ± 1.33)	4 weeks	Overall clinical efficacy, serum inflammatory factor levels
3	Kong Haiying (2025)	RCTs, China	100	45.79 ± 14.16	46.12 ± 13.43	26/24	31/19	Treated with oral mesalazine enteric-coated tablets (0.25 g; heilongjiang tianhong pharmaceutical co., ltd.; national drug approval no. H20103359), one tablet three times daily for 12 weeks	Treated with shaoyao decoction combined with mesalazine	Patients had active mild to moderate UC	12 weeks	Serum inflammatory factor levels, adverse events
4	Shomron Ben-Horin (2024)	RCTs, Israel	41	25 (23–30)	35 (23–48)	5/8	15/13	Received identical-looking mesalazine capsules daily for 8 weeks	Received CurQD: 3 capsules of 500 mg herbal extract and 3 capsules of 500 mg curcumin daily (total 3 g/day) for 8 weeks	Patients had active mild to moderate UC: SCCAI>5, Mayo>2, inflammation>15 cm from rectum	8 weeks	Overall clinical efficacy
5	Zheng Lianlian (2015)	RCTs, China	86	42.3 ± 5.2	41.7 ± 4.9	23/20	25/18	Mesalazine enteric-coated tablets were given	Mesalazine enteric-coated tablets combined with licorice xiexin decoction were given	Diagnosed per the 2007 national IBD consensus with endoscopic and pathological confirmation; condition is mild to moderate	6 weeks	Overall clinical efficacy, serum inflammatory factor levels
6	Shen Lingna (2021)	RCTs, China	60	47.00 ± 10.28	47.67 ± 10.83	15/15	16/14	Oral mesalazine enteric-coated tablets, 4 times/day, 4 tablets per dose (1 g total)	Same mesalazine regimen plus glycyrrhizae decoction (twice daily)	In the traditional Chinese medicine group: 13 cases were mild, 17 were moderate; In the control group: 14 cases were mild, 16 were moderate	8 weeks	Overall clinical efficacy, serum inflammatory factor levels, intestinal microbiota levels, mayo score
7	Hu Lu (2025)	RCTs, China	50	42.33 ± 3.68	42.36 ± 3.64	11/14	13/12	Treated with mesalazine (national drug approval no. H19980148, 0.25 g × 24 tablets), 4 tablets per dose, 3 times per day	Mesalazine enteric-coated tablets combined with licorice xiexin decoction were given	Patients had active mild to moderate UC	NR.	Overall clinical efficacy, serum inflammatory factor levels
8	Li Yan (2024)	RCTs, China	70	45.01 ± 1.61	44.50 ± 1.15	20/15	18/17	Received oral mesalazine enteric-coated tablets (0.25 g, 1.0 g per dose, 3 times/day; H20103359, tianhong pharma)	Mesalazine enteric-coated tablets combined with licorice xiexin decoction were given	Disease severity in the control group: 19 mild cases, 16 moderate; in the experimental group: 21 mild, 14 moderate	8 weeks	Overall clinical efficacy, serum inflammatory factor levels, intestinal microbiota levels, adverse events
9	Feng Yongbo (2023)	RCTs, China	82	39.36 ± 6.25	40.11 ± 6.20	25/16	23/18	Received mesalazine enteric-coated tablets (0.25 g/tablet, 1 g per dose, before meals, 3×/day; H19980148, sunflower pharma)	Mesalazine enteric-coated tablets combined with licorice xiexin decoction were given	Disease duration in the control group ranged from 0.5 to 12 years, with a mean of 5.89 ± 2.11 years; in the experimental group, duration ranged from 1 to 11 years, with a mean of 6.02 ± 1.98 years	8 weeks	Overall clinical efficacy, serum inflammatory factor levels, intestinal microbiota levels
10	Ding Hairong (2018)	RCTs, China	126	42.4 ± 8.7	43.5 ± 9.6	32/31	35/28	Mesalazine granules (Les laboratoires ethypharm, France; batch no. 160117), 1 g per dose, three times daily	Received mesalazine combined with huangqin decoction granules	In the treatment group: 18 mild cases, 35 moderate, 10 severe; In the control group: 21 mild cases, 34 moderate, 8 severe	8 weeks	Overall clinical efficacy, serum inflammatory factor levels, mayo score
11	Wang Haiyang (2024)	RCTs, China	95	49.52 ± 7.13	50.36 ± 7.82	27/20	29/19	Received probiotics plus mesalazine: Bifidobacterium tablets (1.5 g, 3×/day) and mesalazine (1.0 g, 4×/day) for 1 month	Received the same treatment plus huangqin decoction enema	In the control group, there were 26 mild cases and 21 moderate cases of inflammation; in the study group, there were 25 mild cases and 23 moderate cases	4 weeks	Overall clinical efficacy, serum inflammatory factor levels, adverse events
12	Chen Qilong (2019)	RCTs, China	110	20∼60	20∼60	30/25	28/27	Received oral mesalazine alone (500 mg per dose, three times per day)	Treated with oral mesalazine (500 mg per dose, three times per day) combined with retention enema administration of baitouweng decoction	Patients had active mild to moderate UC	8 weeks	Overall clinical efficacy, serum inflammatory factor levels
13	Xu Jiaping (2019)	RCTs, China	124	56.1 ± 7.5	56.3 ± 6.8	30/32	31/31	Received oral mesalazine (produced by harbin zhenikang pharmaceutical co., ltd., brand name: Huidi)	Treated with mesalazine combined with baitouweng decoction administered via enema	In the control group, there were 38 mild cases and 24 moderate cases; in the experimental group, there were 40 mild cases and 22 moderate cases	4 weeks	Overall clinical efficacy, adverse events, mayo score
14	Yang Jingnan (2015)	RCTs, China	46	44.26 ± 12.79	45.83 ± 14.13	11/12	12/11	Mesalazine (500 mg per dose, three times daily) was produced by sunflower pharmaceutical group jiamusi luling co., ltd	Treated with mesalazine combined with baitouweng decoction administered via enema	Patients had active mild to moderate UC	4 weeks	Overall clinical efficacy
15	Chen Min (2022)	RCTs, China	80	40.33 ± 2.64	39.97 ± 2.47	22/18	23/17	Received oral mesalazine (1 g, three times daily; sunflower pharma, H19980148)	Received mesalazine and kangfuxin solution (50 mL; good doctor panxi, Z51021834)	The disease duration was 3 months–6 years (2.65 ± 0.63 years) in the control group and 2 months to 5 years (2.59 ± 0.61 years) in the observation group	4 weeks	Overall clinical efficacy, serum inflammatory factor levels
16	Zhu Qiang (2022)	RCTs, China	46	45.61 ± 3.61	46.02 ± 3.65	13/10	12/11	Received 1 g mesalazine enteric-coated tablets (0.25 g/tablet, 3 times daily)	Received mesalazine and enema treatment with kangfuxin solution (10 mL/bottle)	The disease duration ranged from 1 to 10 years, averaging 5.96 ± 1.05 years in the control group and 6.02 ± 1.08 years in the experimental group	8 weeks	Overall clinical efficacy, serum inflammatory factor levels, mayo score
17	Chen Hui (2022)	RCTs, China	60	35.78 ± 3.14	36.74 ± 3.16	17/13	14/16	Received oral mesalazine enteric-coated tablets (0.25 g/tablet, 1 g per dose, three times daily)	Received mesalazine treatment plus enema with kangfuxin solution (20 mL/bottle)	The disease duration was 1–4 years (2.76 ± 0.11 years) in the control group and 2–5 years (2.79 ± 0.12 years) in the experimental group	4 weeks	Overall clinical efficacy, serum inflammatory factor levels
18	Liu Lina (2022)	RCTs, China	86	47.27 ± 4.27	48.24 ± 4.84	23/20	21/22	Received oral mesalazine (2 g per dose, twice daily) for 2 months	Received mesalazine treatment plus retention enema with kangfuxin solution	Patients had active mild to moderate UC	8 weeks	Overall clinical efficacy, serum inflammatory factor levels
19	Xu Ruosi (2022)	RCTs, China	50	42.03 ± 5.18	41.53 ± 4.68	17/8	16/9	Received mesalazine sustained-release granules (1.0 g per dose, taken with water every 6 h)	Received the same treatment plus mesalazine suppositories and kangfuxin solution retention enema	Observation group: 5 severe, 13 moderate, 7 mild cases; in the control group: 5 severe, 12 moderate, 8 mild cases	4 weeks	Overall clinical efficacy, mayo score, serum inflammatory factor levels
20	Liu Lu (2024)	RCTs, China	80	51.23 ± 11.78	51.31 ± 12.05	20/20	21/19	Received oral mesalazine enteric-coated tablets (1 g per dose, three times daily) for 4 weeks	Received mesalazine treatment plus kangfuxin solution retention enema	Disease duration in the observation group ranged from 7 months to 7 years, with a mean of 3.68 ± 2.77 years; in the control group, it ranged from 6 months to 8 years, with a mean of 3.89 ± 2.98 years	4 weeks	Overall clinical efficacy, serum inflammatory factor levels, adverse events
21	Wang Xudong (2025)	RCTs, China	80	45.21 ± 11.69	45.60 ± 11.87	22/18	23/17	Treated with oral mesalazine enteric-coated tablets (1 g per dose, three times daily) for 4 weeks	Received mesalazine treatment plus retention enema with kangfuxin solution (30 mL kangfuxin +100 mL saline)	Patients had active mild to moderate UC	4 weeks	Overall clinical efficacy, serum inflammatory factor levels, adverse events
22	Liu Yang (2022)	RCTs, China	50	40.24 ± 3.26	40.30 ± 3.22	13/12	12/13	Received oral mesalazine (1.0 g per dose, three times daily)	Received mesalazine treatment plus kangfuxin solution enema	The disease duration was 2 months–5 years (2.63 ± 0.47 years) in the control group and 4 months to 6 years (2.60 ± 0.43 years) in the observation group	4 weeks	Overall clinical efficacy, serum inflammatory factor levels
23	Meng Qinglei (2025)	RCTs, China	78	38.97 ± 5.40	38.67 ± 5.52	22/17	21/18	Received oral mesalazine enteric-coated tablets (0.25 g/tablet, 1.0 g per dose, four times daily) for 4 weeks	Received mesalazine treatment plus kangfuxin solution enema (50 mL)	The disease duration was 3 months to 3 years (1.68 ± 0.52 years) in the observation group and 5 months to 3 years (1.64 ± 0.55 years) in the control group	4 weeks	Overall clinical efficacy, serum inflammatory factor levels, adverse events
24	Yang Ying (2018)	RCTs, China	86	37. 85 ± 7. 06	38. 25 ± 7. 36	25/18	26/17	Received oral mesalazine (0.25 g/tablet, 1 g per dose, four times daily)	Received mesalazine treatment plus oral shenling baizhu powder (6 g per dose, three times daily)	The observation group had a disease duration of 3 months–5 years (mean 8.26 ± 1.25 months); the control group, 4 months to 6 years (mean 8.64 ± 1.74 months)	12 weeks	Overall clinical efficacy, serum inflammatory factor levels, adverse events
25	Dong Zenghui (2018)	RCTs, China	86	46.5 ± 1.5	45.5 ± 2.5	20/23	21/22	Received mesalazine alone (1 g, four times daily) for 12 weeks	Received shenling baizhu powder (6 g, three times daily) plus mesalazine (1 g, three times daily) for 12 weeks with dietary restrictions	Treatment group: disease duration 2–6 weeks, mean 3.5 ± 0.5 weeks; Control group: 1–7 weeks, mean 3.5 ± 0.5 weeks	12 weeks	Overall clinical efficacy, adverse events
26	Li Limei (2021)	RCTs, China	90	46.50 ± 7.87	47.33 ± 8.52	25/20	26/19	Received mesalazine (0.25 g/tablet, dongsheng pharmaceutical co., ltd., approval no. H20020211) at 0.5 g per dose, three times daily	Received mesalazine treatment plus shenling baizhu powder	Observation group: disease duration 8 months to 20 years (mean 9.04 ± 3.24 years); Control group: 6 months to 20 years (mean 8.81 ± 2.15 years)	8 weeks	Overall clinical efficacy, serum inflammatory factor levels
27	Li Kui (2015)	RCTs, China	73	39.5 ± 8.2	40.8 ± 7.6	23/14	21/15	Received oral mesalazine (1.0 g per dose, four times daily; provided by sunflower pharmaceutical group jiamusi luling pharmaceutical co., ltd., approval no. H19980148)	Received mesalazine treatment plus oral shenling baizhu powder (6.0 g per dose, three times daily; provided by Beijing Tongrentang pharmaceutical co., ltd., approval no. Z11020947)	The observation group had a disease duration of 4 months–9 years (mean 9.1 ± 2.7 months); the control group, 5 months to 9 years (mean 9.6 ± 3.1 months)	8 weeks	Overall clinical efficacy, serum inflammatory factor levels
28	Wei Guoli (2013)	RCTs, China	46	18∼58	18∼60	13/10	16/7	Received oral mesalazine, 1.0 g per dose, four times daily	Received the same treatment plus shenling baizhu powder (composed of ginseng, atractylodes, yam, coix seed, etc.), 6.0 g per dose, three times daily	Patients had active mild to moderate UC	12 weeks	Overall clinical efficacy, serum inflammatory factor levels
29	Chen Hong (2014)	RCTs, China	80	39.2 ± 7.6	41.7 ± 8.1	25/15	26/14	Received oral mesalazine enteric-coated tablets (shire, Germany; reg. No. H20030501), 1,000 mg, three times daily	Received mesalazine treatment plus retention enema with shenling baizhu powder (Z51020828, chengdu jiuzhitang jinding pharmaceutical co., ltd.)	The treatment group had a disease duration of 8 months–20 years (mean 8.9 ± 2.4 years); the control group, 10 months to 21 years (mean 9.0 ± 2.6 years)	4 weeks	Overall clinical efficacy
30	He Kuisheng (2014)	RCTs, China	48	37.76 ± 7.12	39.36 ± 6.23	15/9	16/8	Mesalazine (0.25 g/tablet, 1 g per dose, four times daily; sunflower pharmaceutical group jiamusi luling pharmaceutical co., ltd., approval no. H19980148)	Mesalazine and shenling baizhu powder (6 g per dose, three times daily; chongqing greenforest pharmaceutical co., ltd., approval no. Z20053891)	Patients had active mild to moderate UC	13 weeks	Overall clinical efficacy
31	Chen Guozhen (2013)	RCTs, China	96	41.7 ± 8.1	38.2 ± 7.6	24/24	24/24	Treated with mesalazine (Les laboratoires servier, France; H19980149, 10 sachets/box), 1 g per dose, four times daily	Received combined treatment with shenling baizhu powder (3 g/sachet; shaanxi panlong pharmaceutical group co., ltd., Z22021157) and mesalazine	The treatment group had a disease duration of 10 months–18 years (mean 10.2 ± 2.4 years); the control group, 1–20 years (mean 11.0 ± 2.6 years)	8 weeks	Overall clinical efficacy
32	Huang Xiangchun (2016)	RCTs, China	100	42.5 ± 8.5	41.5 ± 8.4	33/17	35/15	Treated with mesalazine (1.0 g per dose, four times daily, orally) for 12 weeks, using mesalazine from heilongjiang tianhong pharmaceutical co., ltd. (approval no. H20103359)	Received the same mesalazine treatment plus shenling baizhu powder	Patients had active mild to moderate UC	12 weeks	Overall clinical efficacy
33	Zhang Aiqing (2019)	RCTs, China	82	39.1 ± 6.4	38.9 ± 6.2	26/16	18/22	Treated with mesalazine enteric-coated tablets (0.5 g per dose, three times daily, taken 30 min after meals; jiamusi luling pharmaceutical co., H19980148)	Received combined treatment with shenling baizhu powder and mesalazine, using the same dosage and administration	The observation group had a disease duration of 1–8 years (mean 4.6 ± 2.5 years); the control group, 1–7 years (mean 4.4 ± 2.3 years)	NR.	Overall clinical efficacy
34	Yang Gong (2012)	RCTs, China	314	44.51 ± 12.02	43.63 ± 12.01	42/38	123/111	Received mesalazine enteric-coated tablets (4 tablets, 4 times/day) + FCC placebo (4 capsules, 3 times/day)	Received fufangkushen colon-coated capsules (4 capsules, 3 times/day) + mesalazine placebo (4 tablets, 4 times/day)	Disease duration: FCC group: 2.99 ± 3.56 years; HD group: 2.39 ± 3.89 years; baseline mayo score: FCC group: 7.86 ± 1.59; HD group: 7.95 ± 1.29	8 weeks	Overall clinical efficacy, adverse events, mayo score

### Network geometry, consistency, and bias assessment

**FIGURE 2:**
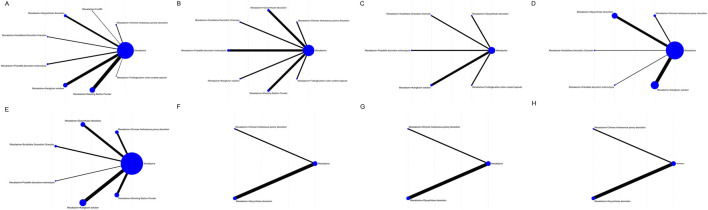
Network plots for eight TCM–mesalazine combinations and mesalazine monotherapy across study outcomes. A–H correspond to: **(A)** clinical efficacy, **(B)** adverse events, **(C)** Mayo score, **(D)** serum IL-6, **(E)** serum TNF-α, **(F)** intestinal Bifidobacteria, **(G)** intestinal Lactobacilli, and **(H)** intestinal *Escherichia coli*.

Networks for all outcomes (Figures 2A–H) were well connected via mesalazine. Contribution plots indicated that comparisons involving Kangfuxin and Shaoyao contributed the largest information. Comparison-adjusted funnel plots showed no clear small-study effects; formal tests were not significant (all *p* > 0.05).

### Clinical efficacy

**FIGURE 3:**
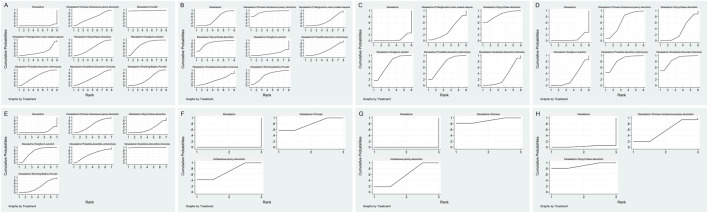
SUCRA rankings of interventions for each outcome **(A–H)**.

**FIGURE 4:**
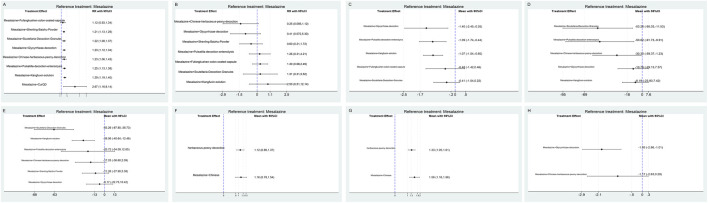
Forest plots of network estimates for each outcome. Data are presented as RRs with 95% CIs for dichotomous outcomes and MDs for continuous outcomes **(A–H)**.

**FIGURE 5:**
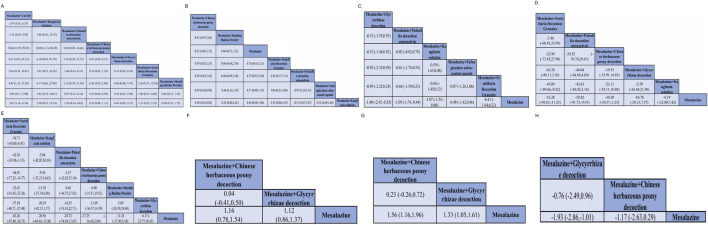
Rankograms for the probability distribution of each intervention’s ranking **(A–H)**.

The comparative efficacy rankings are summarized in Figure 3. Most mesalazine–TCM combinations achieved higher clinical efficacy than mesalazine alone (Figures 4A, 5A). CurQD and Kangfuxin ranked highest by SUCRA (96.7% and 72.7%); in the direct CurQD–mesalazine comparison the pooled effect was RR = 2.67 (95% CI 1.16–6.14; RR > 1 favors the combination). Kangfuxin showed consistent benefit across multiple trials (RR = 1.29, 95% CI 1.19–1.40).

#### Safety profile

Adverse-event rates were similar across regimens (Figures 4B, 5B). Safety rankings favored Shaoyao (SUCRA 90.2%) and Glycyrrhizae (82.1%); no combination showed a statistically significant increase in adverse events.

#### Mayo score reduction

Six interventions reported Mayo score (Figures 4C, 5C). The greatest mean reductions were observed with Glycyrrhizae decoction (MD = −1.40, 95% CI −2.45 to −0.35), Baitouweng decoction via retention enema (MD = −1.09, 95% CI −1.74 to −0.44), and Kangfuxin solution (MD = −1.07, 95% CI −1.54 to −0.60). SUCRA rankings were consistent with these estimates.

#### Inflammatory cytokines

For IL-6 (Figure 4D), the largest decreases were with Scutellaria decoction (MD = −53.28 pg/mL, 95% CI −95.03 to −11.53) and Baitouweng decoction (MD = −50.82 pg/mL, 95% CI −91.73 to −9.91). For TNF-α (Figure 4E), Scutellaria decoction ranked highest (MD = −53.26 pg/mL, 95% CI −87.80 to −38.73), followed by Kangfuxin solution (MD = −26.56 pg/mL, 95% CI −40.64 to −12.48).

#### Intestinal microbiota

Microbiota outcomes were reported in fewer trials (Figures 4F–H, 5F–H).

*Bifidobacteria* increased with Shaoyao (MD = 1.12, 95% CI 0.86–1.37) and Glycyrrhizae (MD = 1.16, 95% CI 0.78–1.54).

*Lactobacilli* increased with Shaoyao (MD = 1.33, 95% CI 1.05–1.61) and Glycyrrhizae (MD = 1.56, 95% CI 1.16–1.96).

*Escherichia coli* decreased significantly with Glycyrrhizae (MD = −1.93, 95% CI −2.86 to −1.01); Shaoyao showed a non-significant trend toward reduction (MD = −1.17, 95% CI −2.63 to 0.29).

Overall, combinations most frequently appearing in the top-three rankings were: CurQD and Kangfuxin for clinical efficacy; Shaoyao and Glycyrrhizae for safety; Glycyrrhizae, Baitouweng, and Kangfuxin for Mayo score reduction; Scutellaria for IL-6 and TNF-α; and Shaoyao/Glycyrrhizae for microbiota modulation.

### Sensitivity analysis for single-trial interventions

To assess the potential instability of the SUCRA rankings due to single-trial interventions (e.g., CurQD and Fufangkushen capsules), sensitivity analyses were performed by excluding these interventions and recalculating the rankings for the remaining treatments. The exclusion of CurQD and Fufangkushen capsules did not significantly alter the overall treatment rankings, suggesting that the top-ranked interventions (e.g., Kangfuxin, Shaoyao decoction) maintained their relative positions in the network. These results indicate that the overall rankings are robust, although caution is recommended when interpreting the rankings for single-trial interventions.

## Adverse events

Adverse events were generally mild and infrequent across all treatment regimens. The most commonly reported adverse events were gastrointestinal discomfort, including symptoms such as bloating, nausea, and diarrhea. These occurred in approximately 10%–15% of patients receiving Kangfuxin solution and Shenling Baizhu Powder. Allergic reactions, such as skin rashes and pruritus, were reported in 2%–3% of patients treated with Fufangkushen capsules. Renal safety was closely monitored, and mild, transient increases in serum creatinine were observed in 1%–2% of patients receiving Kangfuxin solution and Shenling Baizhu Powder. However, these changes were not clinically significant.

The safety profiles of mesalazine–TCM combinations were generally favorable, with most adverse events being mild and self-limiting. Gastrointestinal discomfort was the most common side effect, particularly with Kangfuxin solution and Shenling Baizhu Powder, where 10%–15% of patients reported mild nausea and bloating. Allergic reactions, such as skin rashes and pruritus, were more frequently observed in patients receiving Fufangkushen capsules (2%–3%). Renal function was monitored throughout the study, with mild, transient elevations in serum creatinine noted in 1%–2% of patients treated with Kangfuxin solution and Shenling Baizhu Powder. These changes resolved with dose adjustments. Liver function tests revealed no significant hepatotoxicity, although mild elevations in liver enzymes were observed in some patients receiving Glycyrrhizae decoction.

Overall, the incidence of serious adverse events (SAEs) was low, and mesalazine–TCM combinations were well tolerated. However, individualized monitoring of gastrointestinal, renal, and hepatic function is recommended, particularly for patients with preexisting conditions or those requiring long-term therapy.

## Discussion

This network meta-analysis synthesized evidence from 34 RCTs involving eight standardized TCM formulations combined with mesalazine versus mesalazine monotherapy for mild-to-moderate active UC. Most combinations improved clinical efficacy without increasing adverse events. CurQD formula and Kangfuxin solution ranked highest for clinical response, while Shaoyao decoction and Glycyrrhizae decoction ranked highest for safety. Glycyrrhizae decoction, Baitouweng decoction, and Kangfuxin solution were most effective for Mayo score reduction. Scutellaria decoction showed the greatest improvement in both IL-6 and TNF-α, followed by Kangfuxin for TNF-α. Shaoyao decoction and Glycyrrhizae decoction had the most favorable effects on gut microbiota, with Glycyrrhizae decoction uniquely reducing *E. coli*. These findings suggest that adding selected TCM formulations to mesalazine may provide additive or complementary benefits, and that the optimal choice may depend on the primary clinical goal—symptom remission, inflammation control, or microbiota modulation.

### Comparison with previous evidence

Our finding that Kangfuxin solution ranks among the most effective regimens for clinical response aligns with previous conventional meta-analyses, which have shown that *Periplaneta americana* extract accelerates mucosal healing and improves symptom control in UC (Li et al., 2018; He et al., 2023).

Consistent with earlier safety reviews of these herbal interventions in gastrointestinal disease (Jiabao et al., 2023), none of the TCM–mesalazine combinations increased adverse event rates. Shaoyao decoction and Glycyrrhizae decoction ranked highest for safety, which may reflect their long-standing clinical use and generally mild adverse reaction profile.

Glycyrrhizae decoction and Baitouweng decoction were among the most effective interventions for Mayo score improvement, consistent with their traditional indications for damp-heat syndromes and inflammatory bowel disorders. The observed benefits are plausibly linked to suppression of mucosal inflammation and promotion of epithelial regeneration (Luo et al., 2022).

Scutellaria decoction showed the greatest reductions in IL-6 and TNF-α, supporting pharmacological evidence that Scutellaria baicalensis constituents (e.g., baicalin, baicalein) inhibit NF-κB signaling and downstream proinflammatory cytokine production (Liao et al., 2021).

### Potential mechanisms

The differential efficacy observed among the eight TCM formulations likely reflects their distinct pharmacological profiles and mechanisms of action. Kangfuxin solution contains peptides, nucleotides, and other bioactive compounds that promote epithelial proliferation, angiogenesis, and tissue repair, contributing to its strong performance in mucosal healing (Shu, 1999). Shaoyao decoction, rich in paeoniflorin and glycyrrhizin, exerts anti-inflammatory effects by downregulating proinflammatory cytokines such as IL-6 and TNF-α (Zheng, 2013; Zhen et al., 2024). Glycyrrhizae decoction combines the anti-inflammatory, antimicrobial, and mucosal-protective actions of glycyrrhizin, berberine, and flavonoids, which may explain its broad efficacy across clinical, inflammatory, and microbiota-related outcomes (Liang et al., 2015). Scutellaria decoction, with its high content of baicalin and baicalein, potently inhibits inflammatory signaling pathways (Liao et al., 2021). The CurQD formula integrates curcumin’s NF-κB inhibitory effects with other herbal bioactives for potential synergistic anti-inflammatory action. Shenling Baizhu Powder supports gut barrier integrity and modulates intestinal immune responses through its polysaccharide and saponin components (Rao et al., 2022; Zhang et al., 2024). These mechanistic distinctions highlight the value of tailoring TCM selection to individual patient priorities—whether focused on symptom relief, inflammation control, or microbiota modulation.

As summarized in Table 2, each of the eight TCM formulations evaluated in this study contains key bioactive constituents that contribute to their therapeutic effects in ulcerative colitis (UC). These compounds are associated with various mechanisms of action, such as NF-κB inhibition, mucosal repair, anti-inflammatory effects, and gut microbiota modulation, all of which may explain the observed clinical benefits when combined with mesalazine. The multi-targeted mechanisms of these formulations address both systemic inflammation and intestinal barrier integrity, offering a comprehensive approach to managing UC. The diverse pharmacological profiles of these TCM formulations emphasize the potential of integrative therapies for UC, where the combination of TCM and mesalazine could result in enhanced therapeutic outcomes through synergistic mechanisms.

**TABLE 2:** Summary of bioactive constituents and mechanistic pathways of TCM formulations.

TCM formulation	Key bioactive constituents	Proposed mechanisms of action
CurQD	Curcumin, quercetin, resveratrol	NF-κB inhibition, anti-inflammatory, antioxidant, immunomodulation
Kangfuxin solution	Polypeptides, nucleotides, glycopeptides	Mucosal repair, epithelial proliferation, angiogenesis, intestinal barrier support
Shaoyao decoction	Paeoniflorin, glycyrrhizin	Anti-inflammatory, immune modulation, gut microbiota regulation
Glycyrrhizae decoction	Glycyrrhizin, berberine, flavonoids	Anti-inflammatory, gut microbiota modulation, mucosal protection
Scutellaria decoction	Baicalin, baicalein, scutellarin	NF-κB inhibition, anti-inflammatory, cytokine regulation (TNF-α, IL-6)
Baitouweng decoction	Berberine, paeoniflorin, flavonoids	Anti-inflammatory, antimicrobial (*E. coli* suppression), gut microbiota modulation
Shenling baizhu powder	Polysaccharides, saponins	Gut barrier repair, immune modulation, anti-inflammatory
Fufangkushen capsules	Alkaloids, flavonoids	Anti-inflammatory, immunomodulation, antioxidant properties

## Long-term management of UC and safety considerations

While this study primarily focuses on short-term outcomes, long-term management of UC remains a significant concern. The potential for sustained mucosal healing and relapse prevention with mesalazine–TCM combinations warrants further investigation. While short-term studies suggest efficacy in symptom control and cytokine modulation, longer follow-up is necessary to assess whether these effects translate into durable remission and a reduction in relapse rates. Some formulations, such as Kangfuxin, may promote intestinal epithelial regeneration, which could support mucosal healing over the long term; however, this remains unverified in extended trials.

Additionally, the long-term safety of mesalazine–TCM combinations requires further exploration. Although no significant renal, hepatic, or gastrointestinal toxicities were observed in the short-term studies reviewed, prolonged use may reveal emerging adverse effects. Future studies should include renal function monitoring (e.g., serum creatinine levels), as well as liver and gastrointestinal health assessments, to ensure the safety of long-term combination therapy. Moreover, prospective studies with longer follow-up periods (12–24 months) are necessary to assess relapse rates, sustained remission, and the long-term efficacy of mesalazine–TCM combinations. Given the chronic nature of UC, optimizing long-term disease control while minimizing treatment-associated risks is essential to improving patient outcomes.

### Further exploration of scutellaria Decoction’s anti-inflammatory effects

Scutellaria decoction has demonstrated significant anti-inflammatory effects in the treatment of UC, primarily through the inhibition of NF-κB, a key regulator of inflammation. While our study provides clinical evidence for its efficacy, the precise molecular mechanisms responsible for these effects require further exploration. To validate the hypothesis that Scutellaria decoction exerts its anti-inflammatory effects through NF-κB inhibition, biopsy studies in UC patients could allow for direct molecular analysis of NF-κB activity in colonic tissue. Cytokine profiling, both in serum and local mucosal samples, would help correlate NF-κB inhibition with reductions in proinflammatory cytokines such as TNF-α, IL-6, and IL-1β. These analyses would strengthen the evidence for Scutellaria decoction as a key modulator of inflammation in UC.

Furthermore, molecular pathway analyses, including RNA sequencing and protein expression analysis, could provide deeper insights into additional signaling pathways involved, such as MAPK and JAK-STAT, alongside NF-κB. Identifying the specific pathways modulated by Scutellaria decoction could uncover new therapeutic targets and help explain the variability in patient responses. Personalized treatment strategies, guided by biomarkers of response, could be developed to optimize treatment outcomes. Investigating resistance mechanisms could also shed light on why some patients show suboptimal responses, leading to better patient stratification and more effective therapies. Thus, while this study provides a solid foundation for the clinical efficacy of Scutellaria decoction, future research incorporating biopsy analysis, cytokine profiling, and molecular pathway studies is essential to further validate its mechanisms of action and optimize treatment for UC patients.

### Personalized approach to Mesalazine-TCM combinations based on patient characteristics

Our findings can be translated into a pragmatic, hypothesis-generating framework that matches mesalazine–TCM combinations to patient phenotypes and therapeutic targets. For patients with a high symptomatic burden during active disease, combinations prioritizing rapid clinical response—CurQD and Kangfuxin—ranked most favorably for overall efficacy and are biologically plausible choices given their anti-inflammatory and mucosal-repair profiles. When endoscopic improvement is the principal goal, greater Mayo score reductions with Glycyrrhizae decoction, Baitouweng decoction (notably as a retention enema), and Kangfuxin suggest preferential use in patients with proctitis or left-sided disease, where a topical route may increase local exposure. In a systemic inflammatory phenotype (elevated IL-6/TNF-α at baseline), Scutellaria granules consistently ranked highest for cytokine reduction, with Kangfuxin as a reasonable alternative. For patients with microbiota dysbiosis or frequent relapse, Shaoyao decoction and Glycyrrhizae decoction—which were associated with increases in Bifidobacteria/Lactobacilli and reduced *Escherichia* coli—may be attractive for remission consolidation or maintenance alongside standard mesalazine dosing. Safety signals were broadly comparable to mesalazine alone; nevertheless, clinicians should continue routine renal monitoring for long-term mesalazine, and consider individual intolerance or allergy histories when selecting specific formulas. These subgroup inferences are constrained by study-level (aggregate) data, short treatment windows (3–12 weeks), and incomplete reporting of disease extent and biomarkers across trials; as such, they should be interpreted as exploratory rather than prescriptive. Prospective, biomarker-stratified and route-of-administration–aware RCTs—ideally with individual participant data—are needed to confirm effect modification by disease severity, extent, cytokine profiles, and baseline microbiota, and to determine whether these tailored pairings improve durable remission and relapse prevention.

### Strengths and limitations

This study has several notable strengths. It is the first to apply a frequentist network meta-analysis to directly and indirectly compare eight standardized TCM–mesalazine combinations, incorporating a broad spectrum of outcomes, including clinical efficacy, safety, symptom scores, inflammatory biomarkers, and gut microbiota indices. The use of SUCRA rankings offers a transparent and systematic framework to guide clinicians in selecting adjunctive therapies aligned with specific treatment goals.

However, several limitations should be acknowledged. Most of the included RCTs were single-center studies conducted in China, which may limit the external generalizability of the results. The methodological quality varied across studies, with frequent omissions in reporting critical aspects such as allocation concealment, blinding, and intention-to-treat analyses. Additionally, the follow-up durations were generally short (≤12 weeks), which restricted our ability to evaluate long-term remission, relapse prevention, and sustained mucosal healing. For certain interventions, such as CurQD and Fufangkushen capsules, the evidence stemmed from a single trial, introducing considerable uncertainty into their SUCRA rankings. Moreover, microbiota-related outcomes were assessed in relatively few studies, limiting the robustness of the conclusions in this domain. Addressing these gaps through well-designed, multicenter RCTs with longer follow-up and comprehensive outcome reporting is essential to strengthen the evidence base.

## Clinical implications and future research

The findings suggest that CurQD formula or Kangfuxin solution may be prioritized when the primary objective is short-term clinical remission, as these combinations showed the highest efficacy in rapid symptom relief. In contrast, Glycyrrhizae decoction and Baitouweng decoction may be more appropriate for reducing Mayo scores and promoting mucosal healing. Scutellaria decoction appears particularly effective in lowering systemic inflammatory cytokines, making it a valuable choice for patients with elevated IL-6 or TNF-α levels. Shaoyao decoction and Glycyrrhizae decoction, which help restore gut microbiota balance, may be beneficial in cases of concomitant dysbiosis, contributing to remission consolidation or maintenance when used alongside standard mesalazine dosing.

Future research should include large-scale, multicenter RCTs with rigorous blinding, standardized diagnostic and outcome criteria to allow valid cross-study comparisons. Extended follow-up periods are needed to evaluate the durability of treatment effects, relapse rates, and sustained endoscopic healing. In parallel, mechanistic investigations focusing on immune modulation, epithelial barrier restoration, and microbiota–host interactions will be critical to elucidate the underlying therapeutic pathways and refine patient selection for specific regimens.

## Potential long-term benefits of combining TCM with mesalazine

While this network meta-analysis primarily focuses on short-term clinical outcomes (3–12 weeks), the chronic and relapsing nature of UC warrants consideration of long-term management strategies, particularly regarding relapse prevention and sustained remission. Combining mesalazine with TCM may offer several advantages in long-term UC care:

TCM formulations such as Kangfuxin and Glycyrrhizae decoction have demonstrated potential to reduce inflammation and promote mucosal healing, which may help prevent UC relapses. TCM formulations like Shaoyao decoction could also regulate the gut microbiota and improve intestinal permeability, supporting sustained remission and reducing flare-up frequency. Future studies with longer follow-up are needed to determine if these benefits translate into reduced relapse rates over time.

Given the crucial role of mucosal healing in UC management, TCM formulations that support intestinal epithelial repair (e.g., Kangfuxin) could be beneficial in maintaining mucosal integrity and preventing the progression of UC, including complications like colorectal cancer in patients with longstanding disease. While short-term evidence is promising, long-term studies are essential to confirm the durability of mucosal healing and the potential role of TCM in preventing disease progression.

The potential for nephrotoxicity and gastrointestinal issues with mesalazine highlights the need to ensure the safety of combination therapies. Some TCM formulations, such as Shaoyao decoction, have shown protective effects against gastrointestinal discomfort and liver abnormalities, which may help mitigate the side effects associated with prolonged mesalazine use. However, more studies are needed to assess the safety and tolerability of long-term TCM–mesalazine combinations.

Given the importance of long-term management in UC, further studies with extended follow-up periods (6–12 months or longer) are required to assess the full potential of TCM–mesalazine combinations. These studies should evaluate outcomes such as sustained remission, relapse rates, long-term mucosal healing, and the safety of chronic combination therapy. Future trials should also focus on monitoring renal and hepatic function to ensure the long-term safety of these combination treatments.

### Future insights from advanced microbiota analysis

While microbiota outcomes in this study were based on broad profiling methods with limited taxonomic resolution, the use of 16S rRNA sequencing or metagenomic sequencing would provide more detailed and functional insights into microbial shifts in response to mesalazine–TCM combinations. These advanced techniques would allow for a more comprehensive analysis of microbial species abundance and diversity, as well as the functional capacity of the microbiota, including microbial metabolism and immune modulation.

Higher-resolution analysis through 16S rRNA sequencing could identify specific microbial species—such as Bifidobacteria, Lactobacilli, and *Escherichia* coli—that are directly associated with the pathogenesis of UC and could be modulated by TCM formulations. This approach could help clarify which species are beneficial or harmful in the context of UC and how TCM may alter these populations in ways that support disease remission.

Metagenomic sequencing would provide functional insights into how changes in the microbiota contribute to UC treatment. Specifically, it would allow for the identification of microbial genes and metabolic pathways, such as short-chain fatty acid production and immune modulation, that are crucial for maintaining gut health and reducing inflammation in UC. This approach could uncover microbial mechanisms that directly link to clinical outcomes, such as mucosal healing and symptom relief.

By linking microbial data with clinical outcomes, future studies could identify microbial biomarkers that predict treatment efficacy and resistance to mesalazine–TCM combinations. Understanding these correlations could lead to more targeted and personalized approaches in UC management, ultimately improving treatment outcomes.

## Conclusion

This network meta-analysis of 34 randomized controlled trials provides evidence that combining selected TCM formulations with mesalazine enhances short-term clinical outcomes in mild-to-moderate UC without increasing adverse events. Specifically, the CurQD formula and Kangfuxin solution ranked highest for clinical remission, while Glycyrrhizae decoction and Baitouweng decoction showed the greatest effectiveness for Mayo score reduction. Scutellaria decoction was most effective in suppressing inflammatory cytokines, and both Shaoyao decoction and Glycyrrhizae decoction were particularly beneficial for modulating gut microbiota. The choice of adjunctive regimens should be based on the primary therapeutic goal. However, further high-quality, multicenter randomized controlled trials with extended follow-up are necessary to confirm these findings and provide robust, evidence-based recommendations for clinical guidelines.

## Data Availability

The original contributions presented in the study are included in the article/Supplementary Material, further inquiries can be directed to the corresponding authors.
