# Supplementary material for: Comparative efficacy and safety of mesalazine-based regimens with traditional Chinese medicines in mild-to-moderate ulcerative colitis
Source: Front Pharmacol. 2026 Jan 13;16:1691460. doi: 10.3389/fphar.2025.1691460 (PMC12862138; doi:10.3389/fphar.2025.1691460)
Supplement: Supplementary file 1 [file Supplementaryfile1.docx]

**Supplemental Online Content**

**Comparative Efficacy and Safety of Mesalazine‑Based Regimens with Traditional Chinese Medicines in Mild‑to‑Moderate Ulcerative Colitis**

1. The risk of bias summary for studies included in the meta-analysis.
2. Rankogram.
3. Funnel plot.
4. Contribution plot.
5. Sensitivity analysis
6. **The risk of bias summary for studies included in the meta-analysis.**

**a.**

**
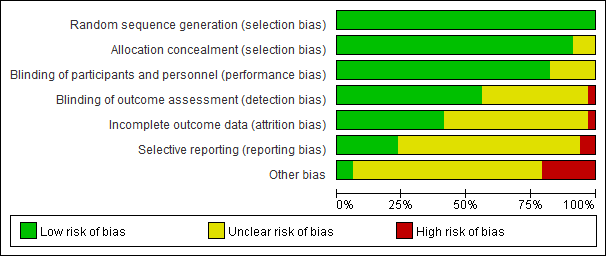
**

Risk of bias graph: review authors' judgements about each risk of bias item presented as percentages across all included studies.

b.


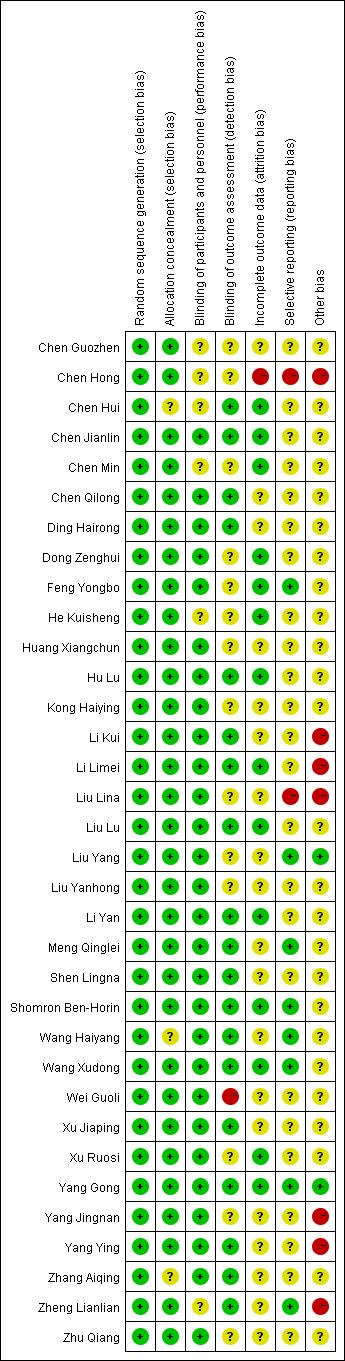


Risk of bias summary: review authors' judgements about each risk of bias item for each included study.

**2. Rankogram.**

**a.**

**b.**

**c.**

**d.**

**e.**

**f.**

**g.**

**h.**

**(a) Clinical efficacy rate; (b)** **Adverse events rate; (c) Mayo score;** **(d) Serum IL-6; (e) Serum TNFa (f); Intestinal Bifidobacteria (g) Intestinal lactic acid bacteria; (h) Intestinal Escherichia coli**

**3. Funnel plot**

**a.**

**b.**

**c.**

**d.**

**e.**

**f.**

**g.**

**h.**

**(a) Clinical efficacy rate; (b)** **Adverse events rate; (c) Mayo score;** **(d) Serum IL-6; (e) Serum TNFa (f); Intestinal Bifidobacteria (g) Intestinal lactic acid bacteria; (h) Intestinal Escherichia coli**

**4. Contribution plot.**

**a.**

**b.**

**c.**

**d.**

**e.**

**f.**

**g.**

**h.**

**(a) Clinical efficacy rate; (b)** **Adverse events rate; (c) Mayo score;** **(d) Serum IL-6; (e) Serum TNFa (f); Intestinal Bifidobacteria (g) Intestinal lactic acid bacteria; (h) Intestinal Escherichia coli**

1. **Sensitivity analysis**


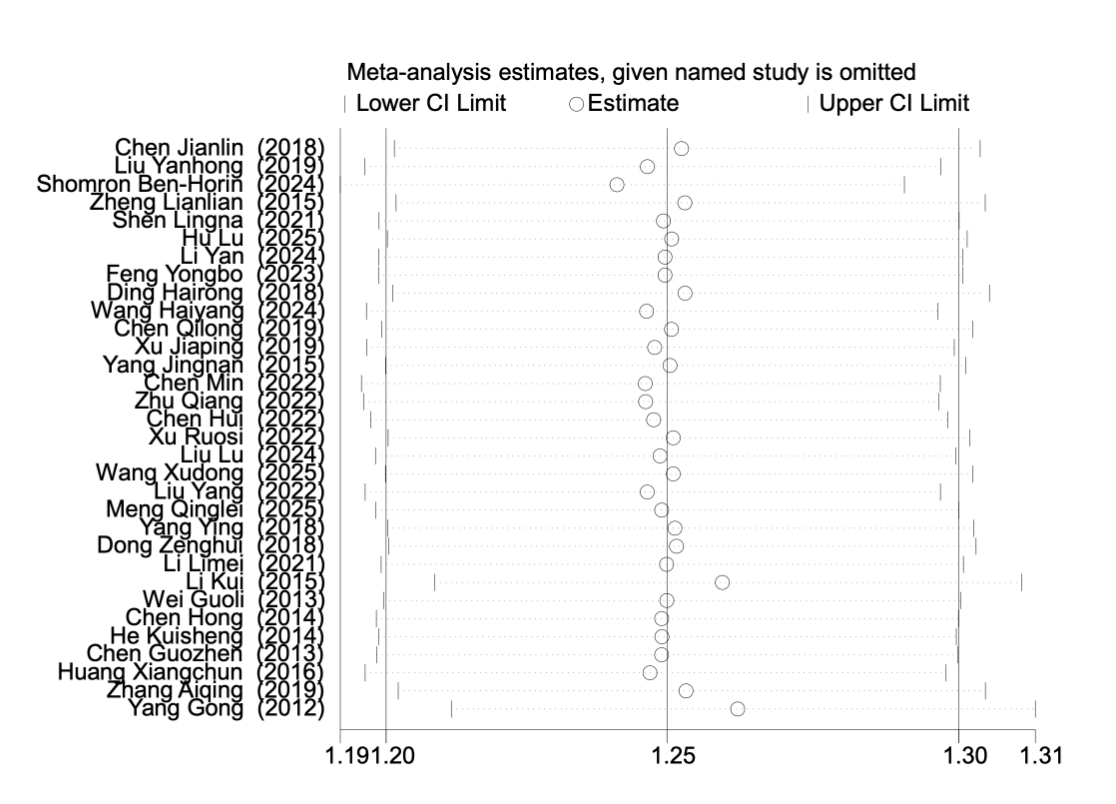


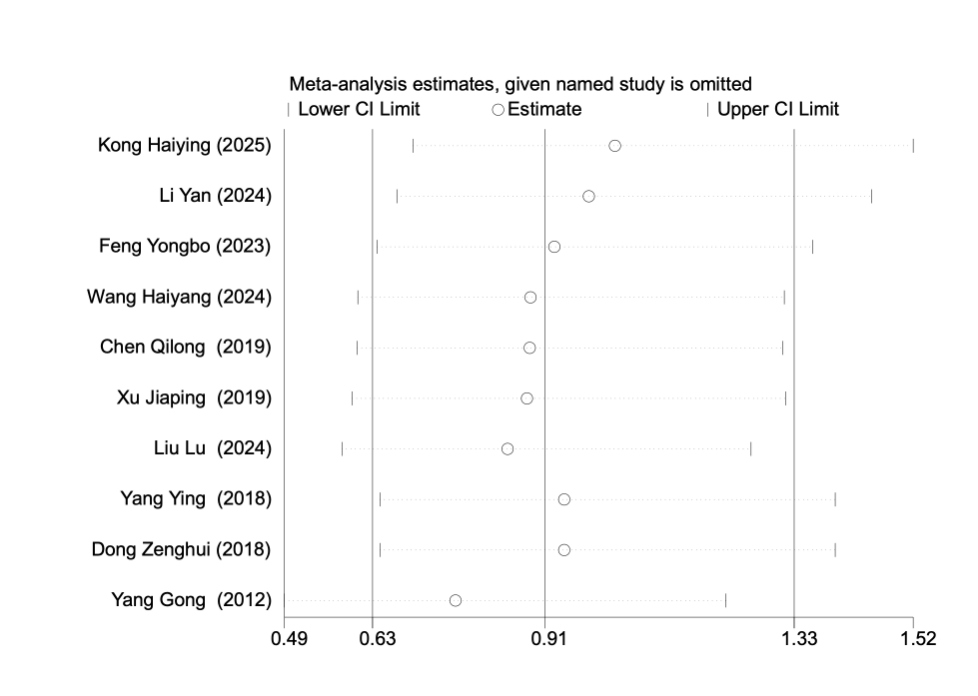


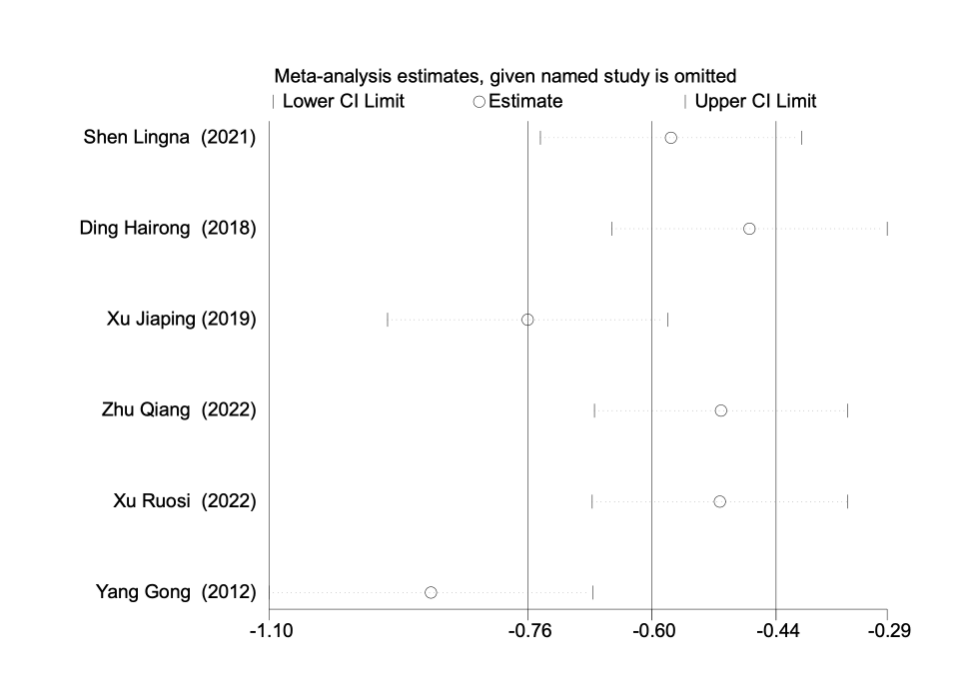


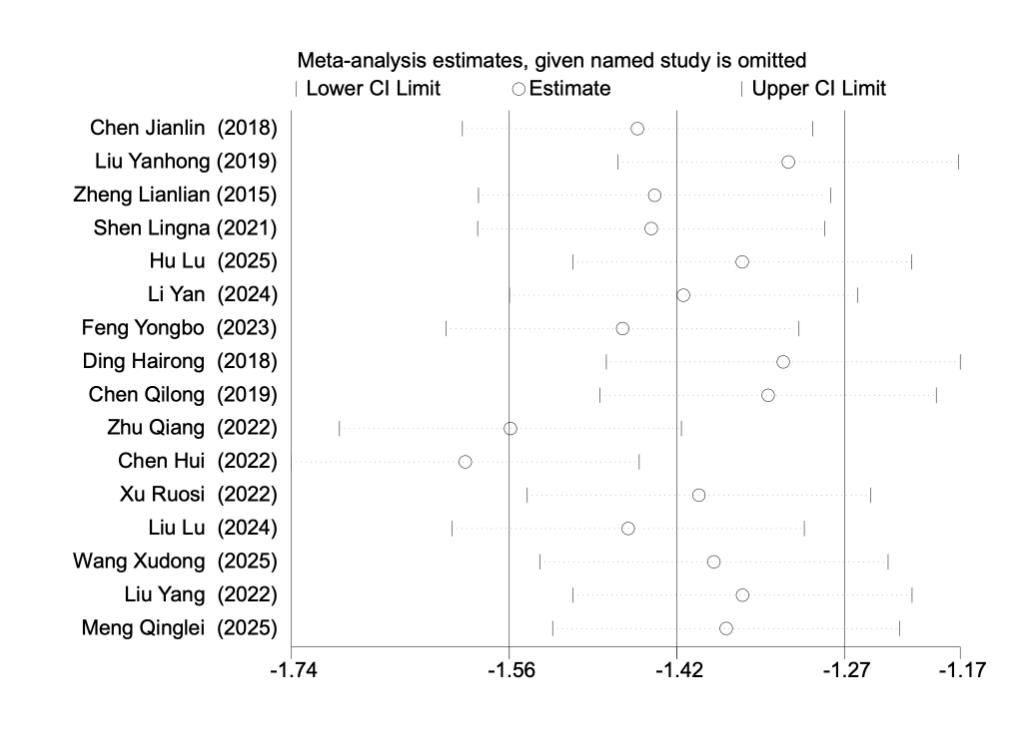


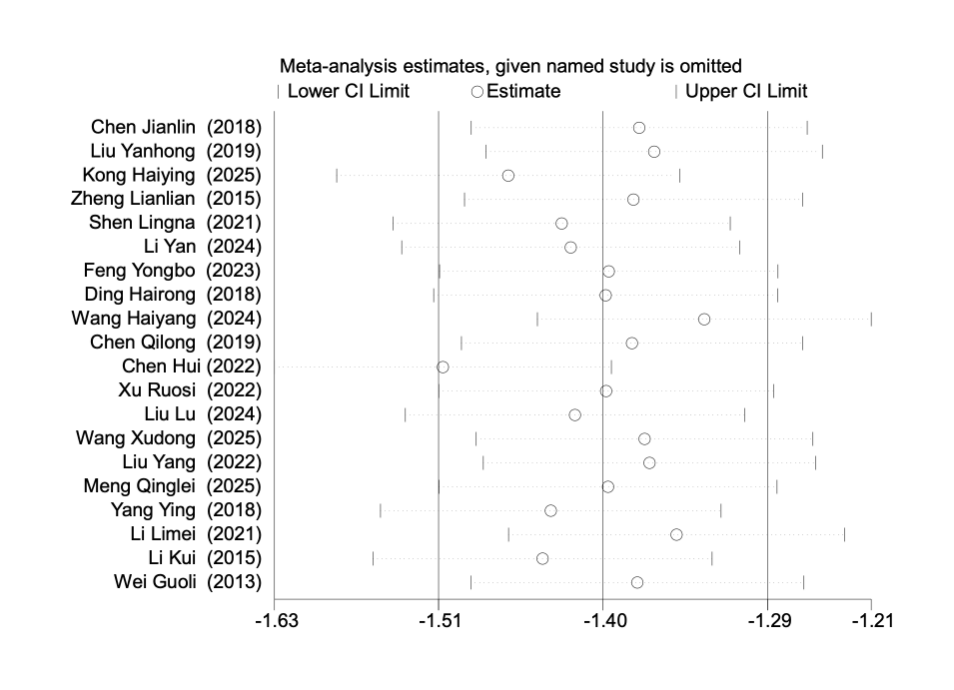


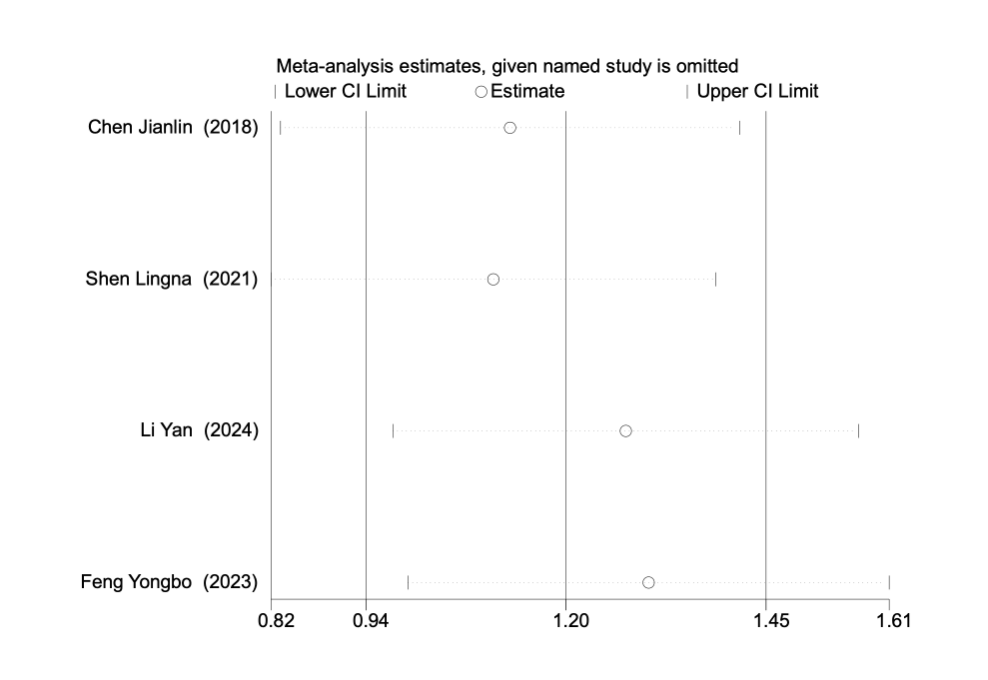


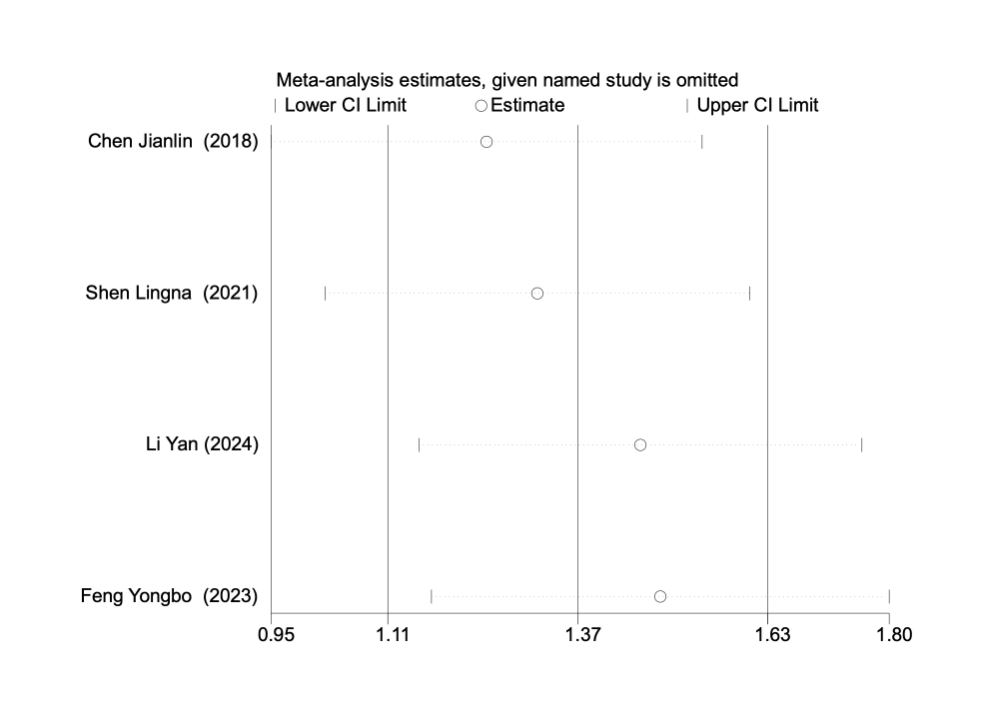


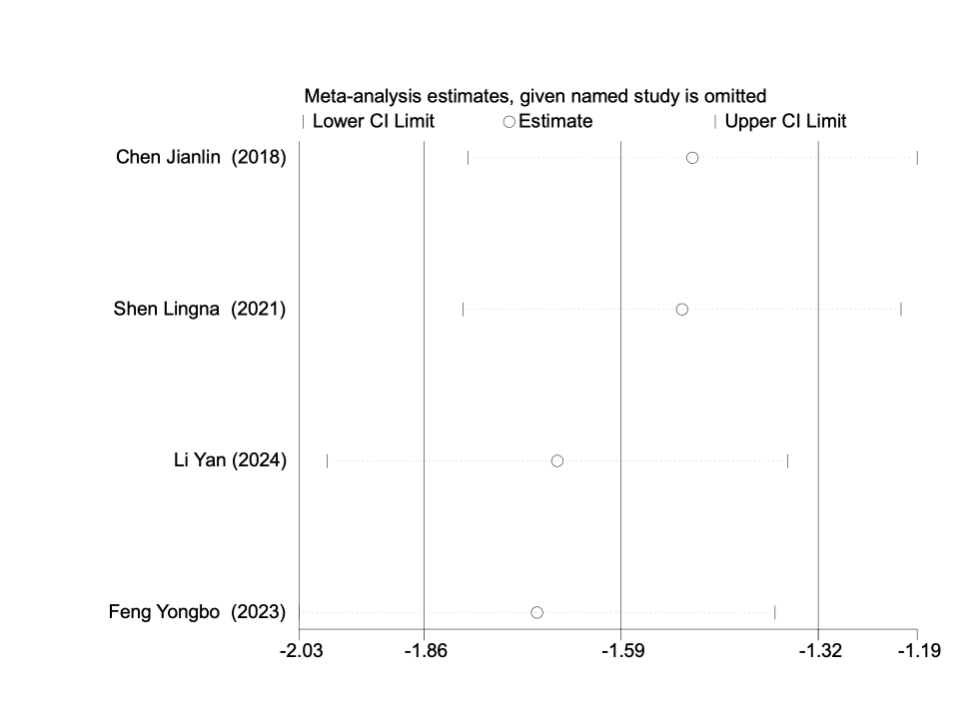


**(a) Clinical efficacy rate; (b) Adverse events rate; (c) Mayo score; (d) Serum IL-6; (e) Serum TNFa (f); Intestinal Bifidobacteria (g) Intestinal lactic acid bacteria; (h) Intestinal Escherichia coli**
